# Mechanism Involved in Acute Liver Injury Induced by Intestinal Ischemia-Reperfusion

**DOI:** 10.3389/fphar.2022.924695

**Published:** 2022-05-23

**Authors:** Binghui Jin, Guangyao Li, Lin Zhou, Zhe Fan

**Affiliations:** ^1^ Department of General Surgery, The Third People’s Hospital of Dalian, Dalian Medical University, Dalian, China; ^2^ Department of Central Laboratory, The Third People’s Hospital of Dalian, Dalian Medical University, Dalian, China; ^3^ Department of Outpatient, the NO. 967 Hospital of PLA Joint Logistics Support Force, Dalian Medical University, Dalian, China

**Keywords:** intestinal ischemia-reperfusion, acute liver injury, mechanism, signal transduction pathways, treatment

## Abstract

Intestinal ischemia-reperfusion (I/R) is a common pathophysiological process, which can occur in many conditions such as acute enteric ischemia, severe burns, small intestinal transplantation, etc,. Ischemia-reperfusion of the intestine is often accompanied by distal organ injury, especially liver injury. This paper outlined the signal pathways and cytokines involved in acute liver injury induced by intestinal I/R: the NF-κB Signaling Pathway, the P66shc Signaling Pathway, the HMGB1 Signaling Pathway, the Nrf2-ARE Signaling Pathway, the AMPK-SIRT-1 Signaling Pathway and other cytokines, providing new ideas for the prevention and treatment of liver injury caused by reperfusion after intestinal I/R.

## 1 Introduction

Intestinal ischemia-reperfusion (I/R) is a series of interrelated pathophysiological processes including vasoconstriction, thrombosis, mitochondrial damage, inflammatory response, cellular damage and cell death. (Hayase et al., 2019). Intestinal I/R is known to occur in severe conditions such as extracorporeal circulation, vascular embolism, and small bowel transplantation. It is a very natural pathophysiological phenomenon (Fan et al., 2019). After intestinal ischemia, the returning blood supply often causes intestinal epithelial sloughing, bacterial shift, and systemic response. In the process, patients often develop systemic inflammatory response syndrome (SIRS) and multiple organ dysfunction syndrome (MODS) (Li et al., 2019a). The gut and the liver are among the most ischemia-sensitive tissues (Camara-Lemarroy, 2014). In addition to damage to the intestines, intestinal I/R has a great impact on other distal organs. When I/R occurs in the intestine, the liver overtakes the intestine as the organ most vulnerable to injury (Jing et al., 2018). The liver is the first organ to be affected, which may because the liver is closest to the gut and competes most with it for blood supply, and its blood vessels are also connected to the intestinal circulation (Hu et al., 2020). The vascular structure of the liver is related to the intestinal cycle, and the hepatoenteric axis plays a vital part in I/R-induced liver injury (Wen et al., 2020). Literature suggests that intestinal I/R disrupts the integrity of the cell membrane, making it a much less effective barrier against invasion by bacteria and inflammatory cytokines (Jia et al., 2020). When the intestinal barrier is compromised, toxins and bacteria in the gut are allowed to enter the portal vein and peripheral circulation, by which time the liver’s defenses have been significantly weakened, further causing the spread of inflammation and exacerbating the condition (Li et al., 2019b).

For our literature search, the Medical Subject Headings (MeSH) terms and key words were as follows: intestinal ischemia-reperfusion, acute liver injury, signal transduction pathways, treatment, and review. How intestinal I/R leads to the inherent mechanism of acute liver injury (Table 1) is not clear, and this review will concentrate on the signal transduction pathways and various cytokines described in articles on acute liver injury published in recent years, including the nuclear factor kappa-B (NK-κB) signaling pathway, P66shc signaling pathway, high mobility group box1 protein(HMGB1) signaling pathway and nuclear factor erythroid 2-related factor 2/AU-rich element(Nrf2-ARE) signaling pathway, explore its potential pathogenesis and applying biomarkers to targeted therapies.

**TABLE 1:** Indexes of acute liver injury in different animal models.

Model	Indexes of acute liver injury
Mice	AST/ALT, TNF-α, IL-6 Ma et al. (2014)
	Level of GSH and the activities of GSH-PX Ma et al. (2014)
	Histopathologic analysis Ma et al. (2014)
Rats	Histopathologic scores Yao et al. (2007), Alexandropoulos et al. (2017), Wen et al. (2020)
	LDH Wen et al. (2020)
	ALT/AST level Yao et al. (2007), Fan et al. (2014), Wen et al. (2020)
	SOD and MPO Yao et al. (2007), Fan et al. (2014)
	ICAM-1, TNF-6, IL-6 Fan et al. (2014)
	TNF-a, IL-6, IL-1b and ICAM-1 Alexandropoulos et al. (2017)
	Level of GSH and the activities of GSH-PX Alexandropoulos et al. (2017)
	MPO activity Yao et al. (2007)

ALT, alanine aminotransferase; AST, aspartate aminotransferase; TNF, tumor necrosis factor; IL, interleukin; GSH, glutathione; GSH-PX, glutathione peroxidase; LDH, lactate dehydrogenase; SOD, superoxide dismutase; MPO. myeloperoxidase; ICAM, intercellular cell adhesion molecule.

## 2 The NF-κB Signaling Pathway

**FIGURE 1:**
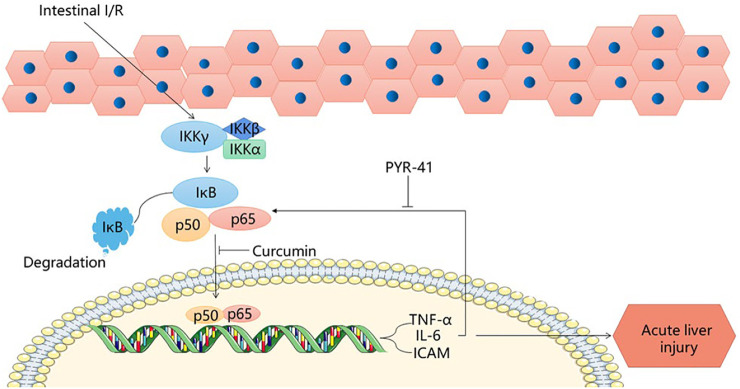
NF-κB signaling pathway.

NF-κB is an important factor in liver injury caused by intestinal I/R. The proteasome, which plays an important regulatory role in signaling, is a multi-catalytic protease complex consisting of two forms, 20 s (700 kDa) and 26 s (2000 kDa). (Bard et al., 2018; Xie et al., 2019a). The initial phase of intestinal I/R occurs when bacterial and endotoxin invasion compromises the intestinal barrier, decreases defenses and immunity, and bacteria, endotoxin, and oxidation stress take the opportunity to enter the circulation and activate NF-κB, causing it to be expressed (Liu et al., 2020). NF-kB translocates to the nucleus (Bellezza et al., 2018), dissociates from IK-κB and acts as an enhancer or repressor to regulate transcription of genes, such as those encoding TNF-a, IL-6, and ICAM-1 (Xie et al., 2019b). By inhibiting the expression of TNF-α and IL-6, curcumin limits the activation of leukocytes in the liver and other tissues, reducing tissue damage caused by inflammatory factors and proteasome activity by inhibiting NF-κB, and inhibiting enhanced ICAM-1 and neutrophil infiltration to protect against liver injury (Fan et al., 2014). After stimulation of macrophages by lipopolysaccharide, the expression of the pro-inflammatory factor TNF-α is initiated. PYR-41 inhibited the stimulation of macrophages by lipopolysaccharide through dose regulation, inhibiting the expression of TNF-α, thereby inhibiting the activation of NF-κB, and reducing the expression of intestinal pro-inflammatory cytokines after I/R (Matsuo et al., 2018). This reduces the damage to the liver after intestinal I/R. MG132 can effectively inhibit the NF-κB pathway to reduce liver injury (Zhao et al., 2010). PYR-41 treatment blocks IκB degradation and activates the NF-κB pathway (Matsuo et al., 20182018). N-acetylcysteine (NAC) and atorvastatin not only protect the liver and kidneys from intestinal I/R injury, but also have a protective effect on the peripheral circulation, as NAC inhibits the release of NF-κB and reduces the production of cytokines TNF- alpha, IL-1 and IL-6, also reducing the damage to the liver in intestinal I/R (Alexandropoulos et al., 2017). The natural antioxidant carnosol can reduce liver injury caused by intestinal I/R by both its own antioxidant action and by inhibiting the NF-κB pathway (Yao et al., 2009). Mangiferin (MF) can reduce NF-κB p65, block NF-κB signaling pathway and protect the liver from post-intestinal I/R damage (El-Sayyad et al., 2017). Therefore, inhibition of the NF-κB pathway is a feasible method to reduce hepatic injury after intestinal I/R. (Figure 1).

## 3 The P66shc Signaling Pathway

**FIGURE 2:**
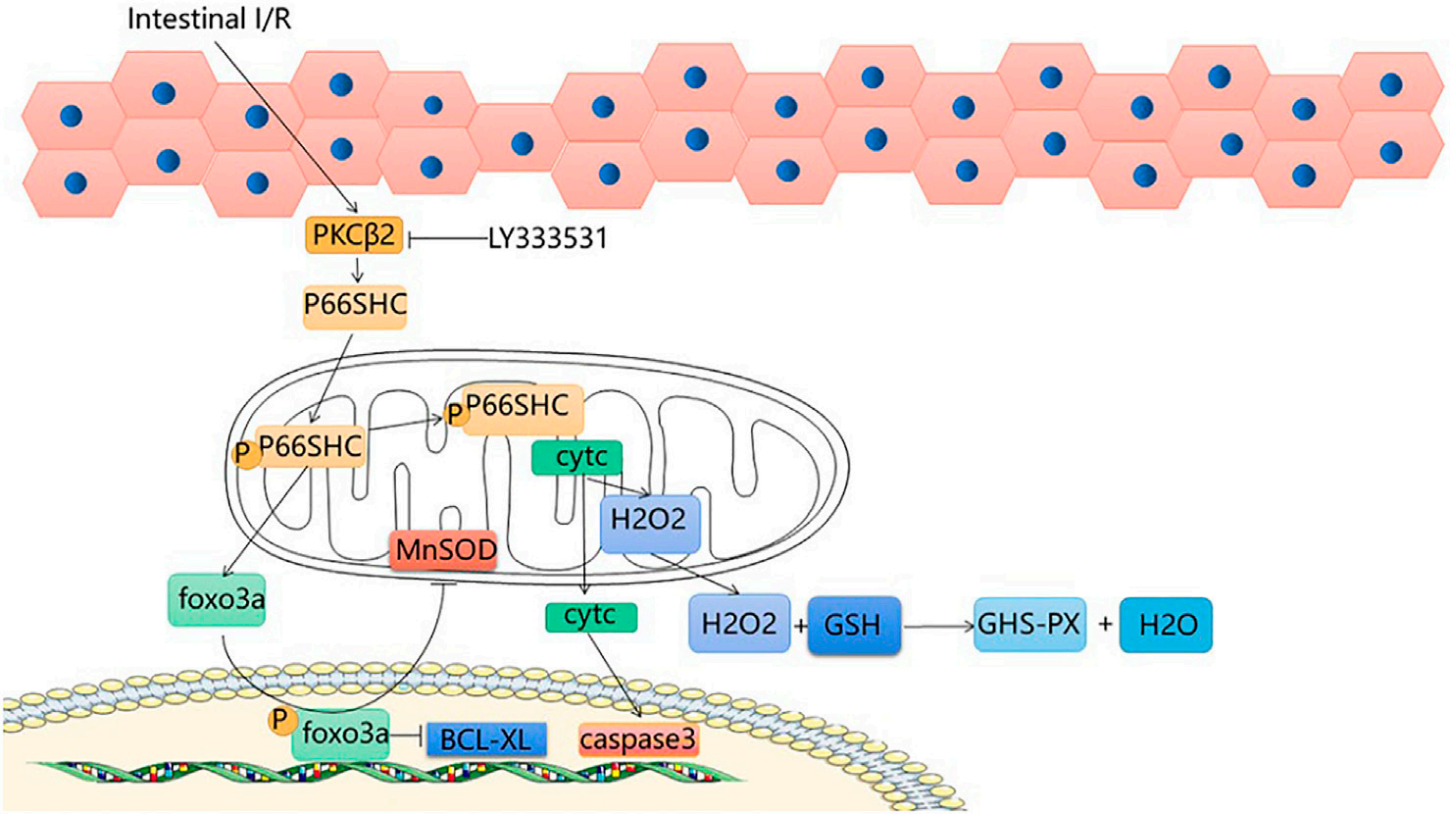
P66shc signaling pathway.

Protein kinase CβII (PKCβII) is a heterodimer of the protein kinase C (PKC) family that is specifically activated during intestinal I/R; after intestinal I/R, oxidative stress activates PKCβII and subsequently the 66 kDa Shc homology 2 domain-containing protein (p66shc) is phosphorylated (Wang et al., 2014). The shc locus can be derived from three isoforms, of which P66SHC is one; it is an oxidative convertase that mediates the production of mitochondrial OS (Zhao et al., 2019). Because of the specific NH2 terminal region, it is able to phosphorylate the serine 36 residue, transferring P66SHC from the cytoplasmic matrix to the mitochondria (Miller et al., 2021). The transfer of p66shc results in a decrease in Ca ion channel responsiveness and an increase in mitochondrial permeability, further enabling excessive OS production and apoptosis (Boengler et al., 2017). Superoxide and peroxide are the main free radicals that cause intestinal I/R. Under normal physiological conditions, endogenous antioxidant enzymes neutralize OS in the body, but when oxygen enters ischemic tissues in large quantities, a large number of free radicals are generated, producing oxidative stress, superoxide dismutase is able to scavenge excess OS and reduce liver damage caused by intestinal I/R, Pistacia lentiscus oil reduces oxidative stress in the liver, reduces tissue damage caused by inflammatory mediators, and reduces liver damage caused by intestinal I/R (Saidi et al., 2017). FOXO3a is a member of the O subclass of the forkhead family and has a function in the regulation of oxidative stress (Baird and Yamamoto, 2020). Manganese superoxide dismutase (MnSOD) can be regulated by FOXO3a (Guo et al., 2021). Phosphop66shc can catalyze the phosphorylation and cytoplasmic translocation of FOXO3a, downgrade MnSOD expression (Fasano et al., 2019), and reduce antioxidant capacity. In a state of cellular oxidative equilibrium, upon release from the mitochondria, cytosolic H_2_O_2_ binds to GSH to produce GSH-PX and H_2_O, and the levels of GSH and GSH-PX can be used as a criterion for the oxidative capacity of hepatocytes (Barlow-Walden et al., 1995; Mailloux et al., 2016). After intestinal I/R, there is excessive production of H_2_O_2_, decreased GSH and inactivation of GSH-PX (Ma et al., 2014), indicators of liver damage. Previous reports have shown that cleaved cystein-3 and BCI-XL can be used as sensitive indicators for the evaluation of p66shc-induced apoptosis (Menini et al., 2006; Cai et al., 2019). Intestinal I/R-induced phosphorylation of hepatic P66shc, leading to upregulation of cleaved-caspase3 and a decrease in BCL-XL, suggesting that intestinal I/R induces liver injury *via* the P66shc pathway. From what has been discussed above, phosphop66shc can catalyze foxo3a phosphorylation and cytoplasm translocation. The p66shc pathway leads to FOXO3a activation, MnSOD downregulation and Bcl-xL expression (Ma et al., 2014). Protocatechuic acid suppresses foxo3a phosphorylation and enhances MnSOD expression. In addition, pro-catecholamines are able to inhibit the upregulation of BCL-XL via p66shc, enhancing the protection of intestinal epithelial cells and hepatocytes against damage caused by intestinal I/R (Ma et al., 2014). In addition, PKCβ plays an important role in the process. The activation of PKCβ-dependent p66shc phosphorylation by intestinal I/R and the cascade reaction of cytochrome-foldase and cystatin-3 activation leads to hepatocyte injury. By giving LY333531, the activation of PKCβ and p66shc phosphorylation was inhibited, and the interaction with cytochrome c was also inhibited, reducing the release of cytochrome c and decreasing the occurrence of apoptosis (Wang et al., 2014). This suggests that inhibition of P66shc phosphorylation may be an effective therapeutic target for liver injury caused by intestinal I/R, and that protocatechuic acid is an effective medicine to treat and alleviate acute liver injury after intestinal I/R. (Figure 2).

## 4 The HMGB1 Signaling Pathway

**FIGURE 3:**
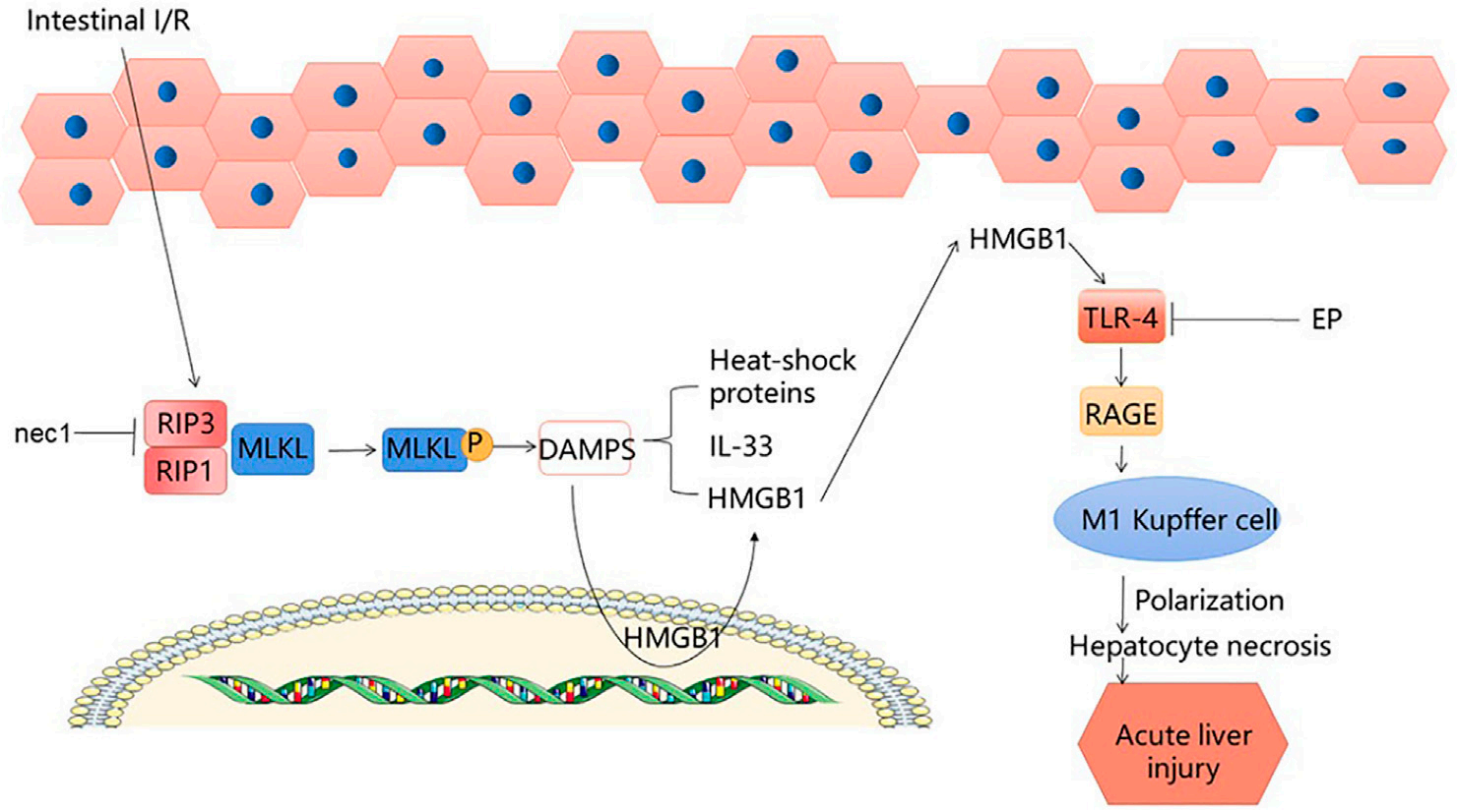
HMGB1 signaling pathway.

When produced extracellularly during cell activation, stress, injury or death, high mobility group box 1 (HMGB1) acts as the ubiquitous histone that tends to cause inflammation (Andersson et al., 2018). Although early inflammatory factors and advanced inflammatory factors are involved in the procedure of intestinal I/R, because the advanced inflammatory factors have a wider treatment time window, HMGB1 has attracted more attention (Wen et al., 2020). According to reports, HMGB1 is released after intestinal I/R injury, thereby triggering the inflammatory response and increasing tissue damage (Linkermann et al., 2013). After intestinal I/R, RIP1 and RIP3 mediate the formation of a necrotic-inducing protein complex, while causing mixed lineage kinase domain-like (MLKL) phosphorylation to greatly increase release of intracellular damage-associated molecular patterns (DAMPs) (Wen et al., 2020). After intestinal I/R, necrotic enterocytes release DAMP, of which HMGB1 is the predominant one (Wen et al., 2017). Catalyzed by endotoxin and endogenous pro-inflammatory cytokines, HMGB1, originally present in the nucleus, can translocate into the cytoplasm (Le et al., 2020). Nec-1 inhibits the RIP1/3 pathway, which in turn inhibits MLKL phosphorylation and reduces HMGB1 translocation from the cytoplasm, leading to a reduction in liver injury. HMGB1-neutralizing antibodies and EP inhibit TLR4 and RAGE expression, thereby reducing the damage to the liver caused by intestinal I/R. There are a large number of single Kupffer cell (KC)s in the liver, which can respond quickly to oxidative stress injury and are stable sentinel cells (Jenne and Kubes, 2013; Ye et al., 2020). At present, there are different theories about how macrophages cause liver damage; however, we found that after intestinal I/R liver injury, the number of KCs increased, and M1-type macrophages dominated. HMGB1 neutralization effectively reduces the polarization of KCs towards M1, and increases the number of M2-type macrophages (Sica and Mantovani, 2012), which reduces liver damage to a certain extent. Uric acid (UA) is a molecule produced by the metabolism of DNA and purines through the xanthine oxidase (XO) pathway (El Ridi and Tallima, 2017). In response to inflammation, HMGB1, a DAMP closely associated with distal organ damage, has been shown to be released from endothelial cells following UA induction (Yang et al., 2006; Cai et al., 2017). In addition, the experimental results showed that UA could induce the release of HMGB1 (Khazoom et al., 2020), increasing liver injury after I/R. To summarize, HMGB1 plays an important role in liver injury after intestinal I/R, and inhibition of HMGB1 can effectively reduce liver injury. This provides a new idea for the treatment of liver injury after intestinal I/R. (Figure 3).

## 5 The Nrf2-ARE Signaling Pathway

**FIGURE 4:**
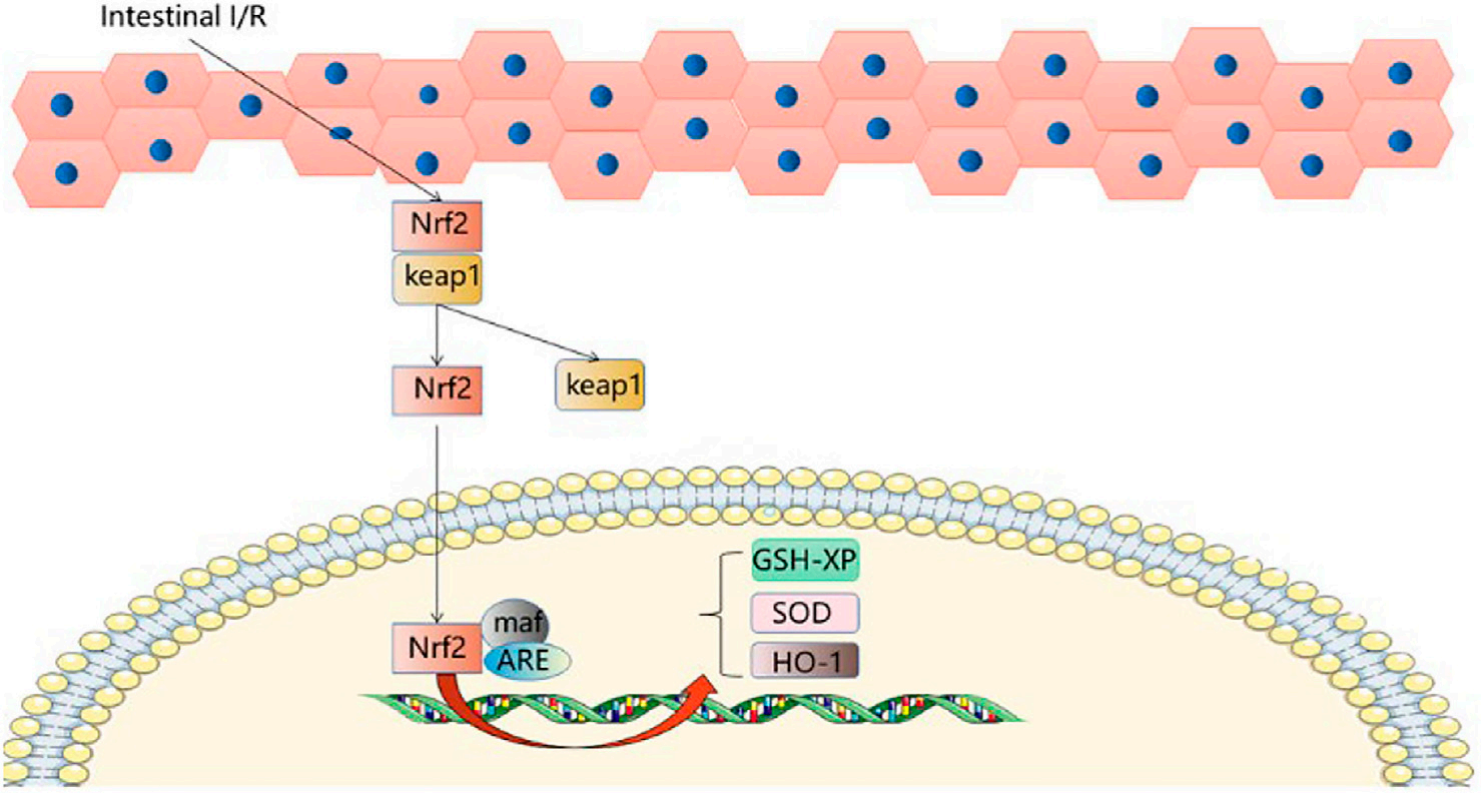
Nrf2-ARE signaling pathway.

The KEAP1-NRF2 pathway plays a major protective role in oxidation and electrophilicity (Camara-Lemarroy, 2014; Bellezza et al., 2018; Baird and Yamamoto, 2020). Under normal conditions, Nrf2 and Keap1 are isolated in the cytoplasm and degraded by the proteasome under basal conditions (Bellezza et al., 2018). When cells are subjected to oxidative stress, the defense mechanism is activated, and NRF2 dissociates from Keap1 and moves to the nucleus to bind with maf, coordinating with the upregulation of protective genes (Hassanein et al., 2020). Nrf2 dissociates from Keap1, translocates from the cytoplasm to the nucleus and binds ARE, activating the expression of hepatic detoxification genes (Jeong et al., 2006; Hassanein et al., 2020), and thereby reducing the reperfusion injury to the liver. Nrf2, activated by sulforaphane, initiates protection of the liver and reduces damage caused by intestinal I/R; both GSH-PX and HO-1 provide endogenous protection for hepatocytes after oxidative stress. Glutamine can upregulate SOD, GSH and GPx levels after II/R, improve the liver’s ability to respond to antioxidant damage and reduce liver damage after II/R (Hartmann et al., 2017). The SFN-treated Nrf2-ARE pathway was able to upregulate GSH-Px and HO-1 (Zhao et al., 2010). SFN improves the protection of the liver by activating the Nrf2-ARE pathway. These are novel targets for prevention and treatment of acute liver injury after intestinal I/R. (Figure 4).

## 6 The AMPK-SIRT-1 Signaling Pathway

**FIGURE 5:**
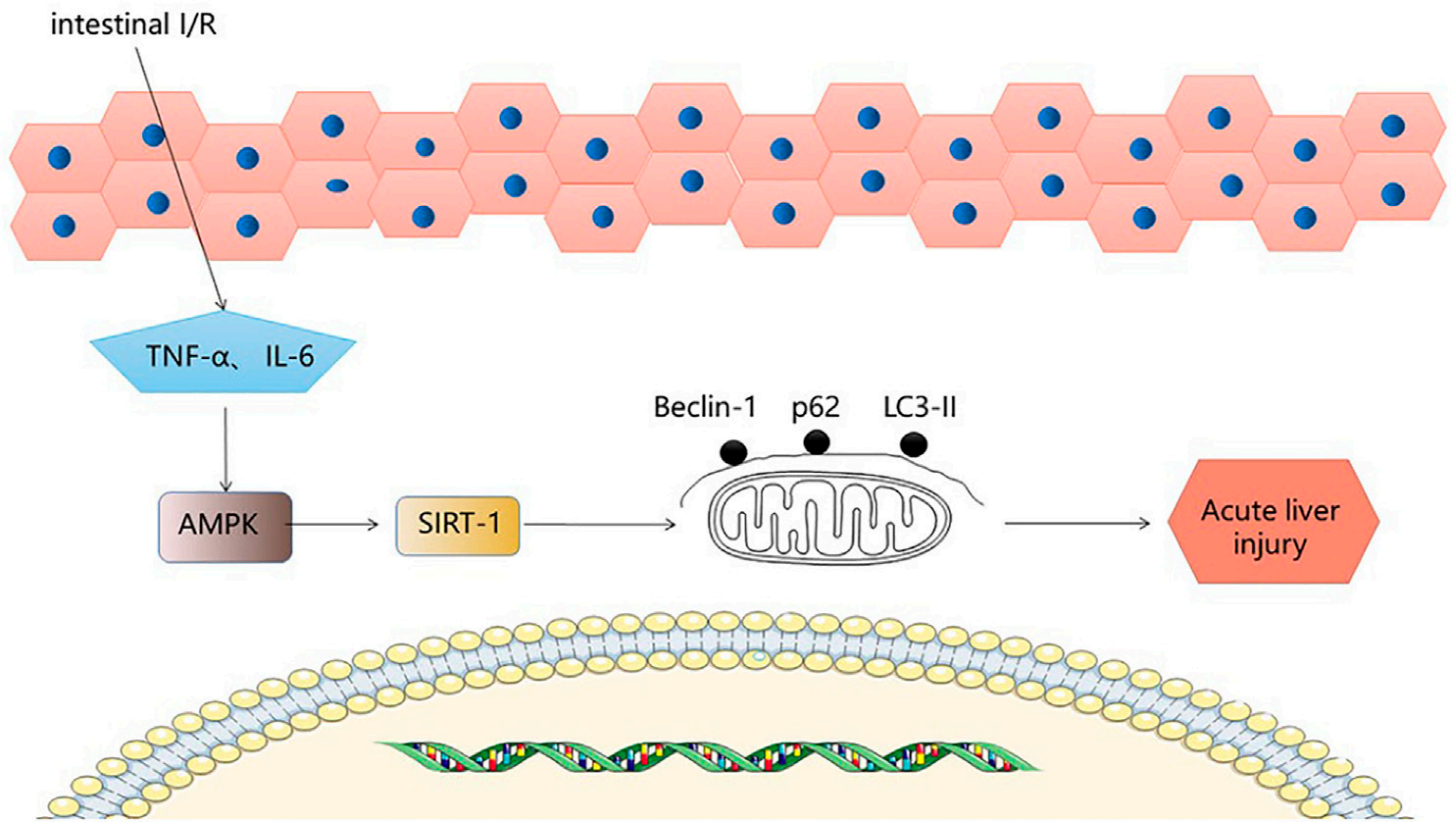
AMPK-SIRT-1 signaling pathway.

Sirtuin1 belongs to the mammalian sirtuin family, which regulates mammalian cell energy and lifespan and is a highly conserved nicotinamide adenine dinucleotide (NAD^+^) dependent, deacetylated family (Meng et al., 2020). Adenosine 5′-monophosphate (AMP)-(9) activated protein kinase is a cellular stress sensor involved in I/R. It has been shown to enhance sirT-1 activity by increasing cellular NAD + levels. Autophagy is a pathological phenomenon involving the degradation of lysosomes and is specific to eukaryotic cells (Shi et al., 2019). Autophagy is a highly conserved catabolic process, usually induced under stressful conditions, that protects cells from damage. During autophagy, autophagosomes engulf cytoplasmic components while the cytoplasmic form of LC3-I binds to phosphatidylethanolamine to form LC3-II in a continuous ubiquitination reaction, and LC3-II within the autophagosome is degraded, so that intracellular LC3-II can represent autophagic activity (Tanida et al., 2008). P62 is widely distributed in the cytoplasm and nucleus as well as in autophagosomes and lysosomes. During oxidative stress, it is translocated to autophagic substrates. Autophagy is the main cause of P62 degradation and autophagic damage is accompanied by a large accumulation of P62, therefore P62 can also represent autophagic activity (Katsuragi et al., 2015). Beclin1 inhibits vesicle processing in the late stages of the autophagic cascade, leading to further cell death, and therefore Beclin1 abundance can be used as an important indicator of autophagic activity (Shi et al., 2019). Therefore, LC3Ⅱ, P62/SQSTM1, and Beclin 1 are key proteins in the autophagic process. Moderate autophagy plays a protective role. After intestinal I/R stimulation, the expression of basic autophagy regulatory factors in rat liver decreased (Beclin 1 and LC3-II decreased, P62 increased) (Jing et al., 2018). Hepatic tissue edema, liver dysfunction and reduced expression of autophagy-related p-AMPK/AMPK/SIRT-1 protein and mRNA provide evidence for a role of AMPK/SIRT-1/autophagy in liver injury due to intestinal I/R. The expression of three key autophagy proteins was found to be increased in the liver after I/R stimulation following Fish oil (FO) induction. Phosphorylation of AMPK is regulated by oxidative stress (Wang et al., 2018), and AMPK phosphorylation can regulate SIRT-1 activity (Jing et al., 2014). In addition, p-AMPK/AMPK and SIRT-1 protein expression decreased in the liver after intestinal I/R, but after FO induction, pAMPK/AMPK and SIRT-1 protein expression increased, with results paralleling autophagy levels. The experimental results confirmed that FO induced autophagy via the AMPK/SIRT-1 signaling pathway in intestinal I/R-induced liver injury, providing new therapeutic ideas for the prevention of intestinal I/R-related liver disease. (Figure 5).

## 7 Other Cytokines

In addition to these pathways, T cells also play a role in regulating I/R injury in the lung, liver and intestine (Funken et al., 2021). The γδ T cells are a unique T cell subpopulation which is one of the earliest developed T cells in all vertebrates (Hayday, 2000; Chien et al., 2014; Dimova et al., 2015). A study found that deficiency in γδ T cells significantly reduced the production of proinflammatory cells and reduced distal organ damage, especially in the liver (Funken et al., 2021). In addition, there is evidence that platelets, in combination with complement and Paneth cell-derived interleukin-17A, can cause liver injury. Formation of histone and neutrophil extracellular traps leads to distant liver injury following intestinal ischemic injury. Blocking the production of histone and neutrophil extracellular traps by recombinant thrombomodulin is an effective way to ameliorate liver injury, thus helping to reduce mortality after intestinal I/R (Hayase et al., 2019). Ethanol is also involved in the mechanism of hepatic injury caused by intestinal I/R. Experimental results show that early intake of low dose ethanol can reduce liver inflammation and damage to liver cell function caused by intestinal I/R. In contrast, high doses of ethanol have the opposite effect, exacerbating liver damage (Yamagishi et al., 2002). IL-17a plays a key role in the development of intestinal I/R injury and subsequent remote hepatic and renal dysfunction. It can also regulate various systemic diseases such as sepsis (Li et al., 2019c; von Stebut et al., 2019). Intestinal I/R causes IL-17A from Paneth cells rapidly to release cytokines such as TNF-α and IL-6, resulting in liver injury. In addition, intestinal I/R inhibited the expression of FXR, PXR and CAR in the liver (Ogura et al., 2012). Moderate dose dexmedetomidine alleviates I/R mediated liver injury by inhibiting NLRP3 inflammasome activation. The gut vascular barrier (GVB) is a separate unit in the intestinal mucosa that blocks the spread of bacteria through the portal vein (Bertocchi et al., 2021). Dexmedetomidine can also act on the GVB/Wnt/β-catenin signaling pathway, upregulates β-catenin, reduces GVB damage, prevents inflammatory mediators from entering the body circulation through the intestinal lumen and then entering the liver, and reduces liver damage in II/R conditions, the exact mechanism of which needs to be further investigated (Zhang et al., 2022). Endothelial nitric oxide synthase (eNOS) produces the well-known vasodilator nitric oxide (NO) (Khalaf et al., 2019), sildenafil reduces liver damage from intestinal I/R by increasing eNOS and increasing NO levels in tissues, thereby dilating blood vessels (Inan et al., 2013). These findings also provide a new direction for the prevention and treatment of liver injury after intestinal I/R injury.

## 8 Conclusion

Intestinal I/R injury is a common type of cell injury that usually occurs after acute intestinal ischemia, small bowel transplantation, and severe burns. Intestinal I/R usually causes distal organ injury. Owing to the special anatomical relationship between the liver and intestine, intestinal I/R often causes acute liver injury. This paper reviews the possible signal pathways of acute liver injury caused by intestinal I/R. Drug regulation of signal pathways can enhance the protection of liver and reduce liver damage, providing a new clinical direction for the prevention and mitigation of liver injury caused by intestinal I/R, and more targeted drugs remain to be discovered.
